# Oral health care during pregnancy to prevent preterm birth and low birth weight: study protocol for a randomized controlled trial

**DOI:** 10.1186/s13063-025-09355-y

**Published:** 2025-12-10

**Authors:** Junko Yasuoka, Yohei Takeshita, Shunsuke Okada, Prodhan MD Rubayet Alam, Akira Shibanuma, Keiko Nanishi, Yoko Sato, Md. Zahid Hossain, Musfiqa Aman, Fahmida Karim, Sultana Razia, Rifat Rezwana, Md Mahmudur Rahman, Nusrat Jahan Khan, Naoki Nakashima, Rafiqul Islam, Kimiyo Kikuchi

**Affiliations:** 1 Division of Global Health Sciences, Graduate School of Public Health, St. Luke’s International University, Tokyo, Japan; 2 Department of Oral and Maxillofacial Radiology, Faculty of Medicine, Dentistry and Pharmaceutical Sciences, Okayama University, Okayama, Japan; 3 Department of Oral and Maxillofacial Radiology, Medical Development Field, Okayama University, Okayama, Japan; 4 Department of Orthodontics, Dental Unit, TMSS Medical College & Rafatullah Community Hospital, Bogura, Bangladesh; 5 Department of Community and Global Health, Graduate School of Medicine, The University of Tokyo, Tokyo, Japan; 6 Office of International Academic Affairs, Graduate School of Medicine, The University of Tokyo, Tokyo, Japan; 7 Department of Health Sciences, Graduate School of Medical Sciences, Kyushu University, Fukuoka, Japan; 8 Department of Periodontology and Oral Pathology, Holy Family Red Crescent Medical College Hospital, Dhaka, Bangladesh; 9 Department of Periodontology, Dental Unit, TMSS Medical College & Rafatullah Community Hospital, Bogura, Bangladesh; 10 Department of Obstetrics & Gynecology, Holy Family Red Crescent Medical College Hospital, Dhaka, Bangladesh; 11 Department of Obstetrics & Gynecology, TMSS Medical College & Rafatullah Community Hospital, Bogura, Bangladesh; 12 Department of Molecular and Cellular Biochemistry, Division of Oral Health, Brain Health and Total Health, Faculty of Dental Science, Kyushu University, Fukuoka, Japan; 13 Global Communication Center, Grameen Communications, Dhaka, Bangladesh; 14 Department of Medical Informatics, Graduate School of Medical Sciences, Kyushu University, Fukuoka, Japan; 15 Division of Healthcare Digital Transformation, Kyushu University, Fukuoka, Japan; 16 Department of Research Promotion, Institute for Asian and Oceanian Studies, Kyushu University, Fukuoka, Japan

**Keywords:** Preterm birth, Low birth weight, Oral health, Periodontal treatment, Chlorhexidine gluconate mouthwash, Randomized controlled trial, Bangladesh

## Abstract

**Background:**

Periodontal disease during pregnancy has been associated with an increased risk of preterm birth (PTB) and low birth weight (LBW). However, most previous interventional studies have reported that scaling and root planing (SRP), a form of basic periodontal treatment, is insufficient to effectively prevent PTB and LBW. Recent systematic reviews and meta-analyses suggest that combining SRP with routine use of chlorhexidine gluconate (CHG) mouthwash may reduce the risk of these adverse birth outcomes by approximately half. This study will be among the first randomized controlled trials (RCTs) to evaluate the potential synergistic effects of this combined approach on improving birth outcomes.

**Methods:**

This multi-center, three-arm RCT will be conducted at two hospitals in Dhaka and Bogura, Bangladesh, between April 2025 and September 2026. A total of 480 pregnant women less than 20 weeks’ gestation attending antenatal checkups will be recruited. Participants diagnosed with periodontal disease will be randomly assigned to either an intervention group or a positive control group. Participants without periodontal disease will be assigned to a negative control group. The intervention consists of SRP combined with continued use of CHG mouthwash throughout pregnancy. The primary outcome will be the rate of PTB. Secondary outcomes include the birth weight of the newborn baby, maternal periodontal status, and oral health-related quality of life (OHRQoL). Effectiveness will be assessed by comparing outcomes across the study groups.

**Discussion:**

The oral health intervention is expected to improve both birth outcomes and maternal oral health. Demonstrating its effectiveness in a real-world setting may support the integration of oral health care into broader maternal, neonatal, and child health strategies, particularly in resource-limited settings.

**Trial registration:**

UMIN Clinical Trials Registry. UMIN000057519. Registered on April 4, 2025. https://center6.umin.ac.jp/cgi-open-bin/ctr/ctr_view.cgi?recptno=R000065719.

**Supplementary Information:**

The online version contains supplementary material available at 10.1186/s13063-025-09355-y.

## Background

More than 60 countries are currently off track to meet the Sustainable Development Goal target of reducing the neonatal mortality rate to 12 or fewer per 1000 live births by 2030 [1, 2]. Among the major contributors to neonatal and infant mortality are preterm birth (PTB), defined as birth before 37 weeks of gestation, and low birth weight (LBW), defined as a birth weight of less than 2500 g [1]. PTB alone accounts for approximately 40% of neonatal deaths [2, 3], while LBW and prematurity together contribute to an estimated 60–80% of global neonatal mortality [4, 5]. Moreover, both PTB and LBW are associated with long-term adverse health outcomes. Infants born preterm are at increased risk of cerebral palsy, cognitive impairment, and developmental abnormalities [6, 7]. Babies born with LBW face higher risks of chronic noncommunicable diseases in adulthood, including cardiovascular diseases, type 2 diabetes, and hypertension [8, 9].

To address these issues, strengthening the continuum of care (CoC) in MNCH services is strongly recommended [10]. The MNCH CoC framework aims to ensure access to essential health services throughout the life course by providing all women with access to reproductive health care before and during pregnancy and childbirth, and by ensuring that newborns receive the care necessary for healthy growth [11]. Systematic reviews and meta-analyses have consistently shown that ensuring CoC is critical for enhancing MNCH outcomes, especially in low- and middle-income countries [12, 13]. Furthermore, recent research has advocated for integrating additional health programs, such as noncommunicable disease prevention and treatment, into the MNCH CoC to strengthen its impact [14].

Emerging evidence suggests that poor oral health, particularly periodontal disease, may adversely affect overall health, including MNCH outcomes such as PTB and LBW [15, 16]. Pregnancy often exacerbates oral health problems due to hormonal changes, especially elevated estrogen and progesterone levels, which impair immune responses and lead to increased vascular permeability and gingival inflammation [17]. A systematic review and meta-analysis of 20 studies found that the global prevalence of periodontal disease among pregnant women is approximately 40% [18].

Epidemiological studies have proposed both direct and indirect pathways through which periodontal disease may contribute to adverse birth outcomes [19–21]. In the direct pathway, periodontal pathogens can reach fetal-placental tissues via hematogenous dissemination, triggering local inflammation and tissue damage that lead to pregnancy complications. In the indirect pathway, inflammatory cytokines and mediators from the gingiva and/or acute-phase reactants from the liver accumulate in the intrauterine compartment, exacerbating inflammation and contributing to pregnancy complications [20, 22]. The adverse impact of periodontal disease on pregnancy outcomes tends to worsen as the severity of maternal periodontal inflammation increases [23, 24].

Both MNCH and oral health require urgent public health attention in Bangladesh. In 2020, Bangladesh had the highest rate of PTB in South Asia at 16.2% [25]. The prevalence of LBW has remained persistently high at 25.7% in 2000, 24.5% in 2010, and 23.0% in 2020 [26]. The most recent figure exceeds that of many other developing countries [1]. The country’s neonatal mortality rate was reported at 20 deaths per 1000 live births in 2022 [27], which is significantly higher than the SDG target. Data on oral health are scarce, particularly in pregnant women, who are at high risk of developing periodontal diseases. A recent cross-sectional study in Dhaka reported that 95.3% of pregnant women had periodontal disease, with 52.4% diagnosed with gingivitis and 43% with periodontitis [28].

Although the relationship between oral health during pregnancy and birth outcomes has garnered increasing attention, effective oral care strategies for the prevention of PTB and LBW remain underexplored. Most interventional studies have found that basic periodontal treatment, which consists of scaling to remove plaque and calculus above the gingival margin and root planing to remove calculus below the gingival margin, is insufficient to prevent PTB and LBW [29, 30]. However, recent systematic reviews and meta-analyses suggest that combining scaling and root planing (SRP) with routine oral care using mouthwash containing chlorhexidine gluconate (CHG) may reduce the risk of PTB and LBW by approximately half (PTB: RR = 0.56; LBW: RR = 0.47) [31]. Another review found that the same combination significantly reduced the risk of PTB (OR = 0.29) and the composite outcome of PTB and/or LBW (OR = 0.18) [32]. The most recent meta-analysis further supported the effectiveness of combining SRP with any mouthwash, reporting a pooled RR of 0.44 for PTB and 0.33 for LBW [33]. Despite these promising results, there remains a lack of well-designed randomized controlled trials (RCTs) to confirm the effectiveness of this combined approach [31–33].

Given the urgent need to establish effective oral health management strategies for pregnant women, integrating oral health care into the MNCH CoC represents a promising and potentially scalable approach. This study therefore aims to (1) assess whether the association between periodontal disease and adverse birth outcomes observed in previous research is also evident in our study population, and (2) evaluate the effectiveness of an integrated intervention, combining SRP and CHG mouthwash use, delivered through MNCH CoC, in preventing PTB and LBW. This RCT will also examine the intervention’s impact on maternal periodontal status and oral health-related quality of life (OHRQoL). Demonstrating the effectiveness of this intervention could provide critical evidence for incorporating oral health into broader MNCH strategies. We believe this study offers a unique and timely contribution to improving MNCH outcomes through an oral health approach.

## Methods

### Study design

**Fig. 1:**
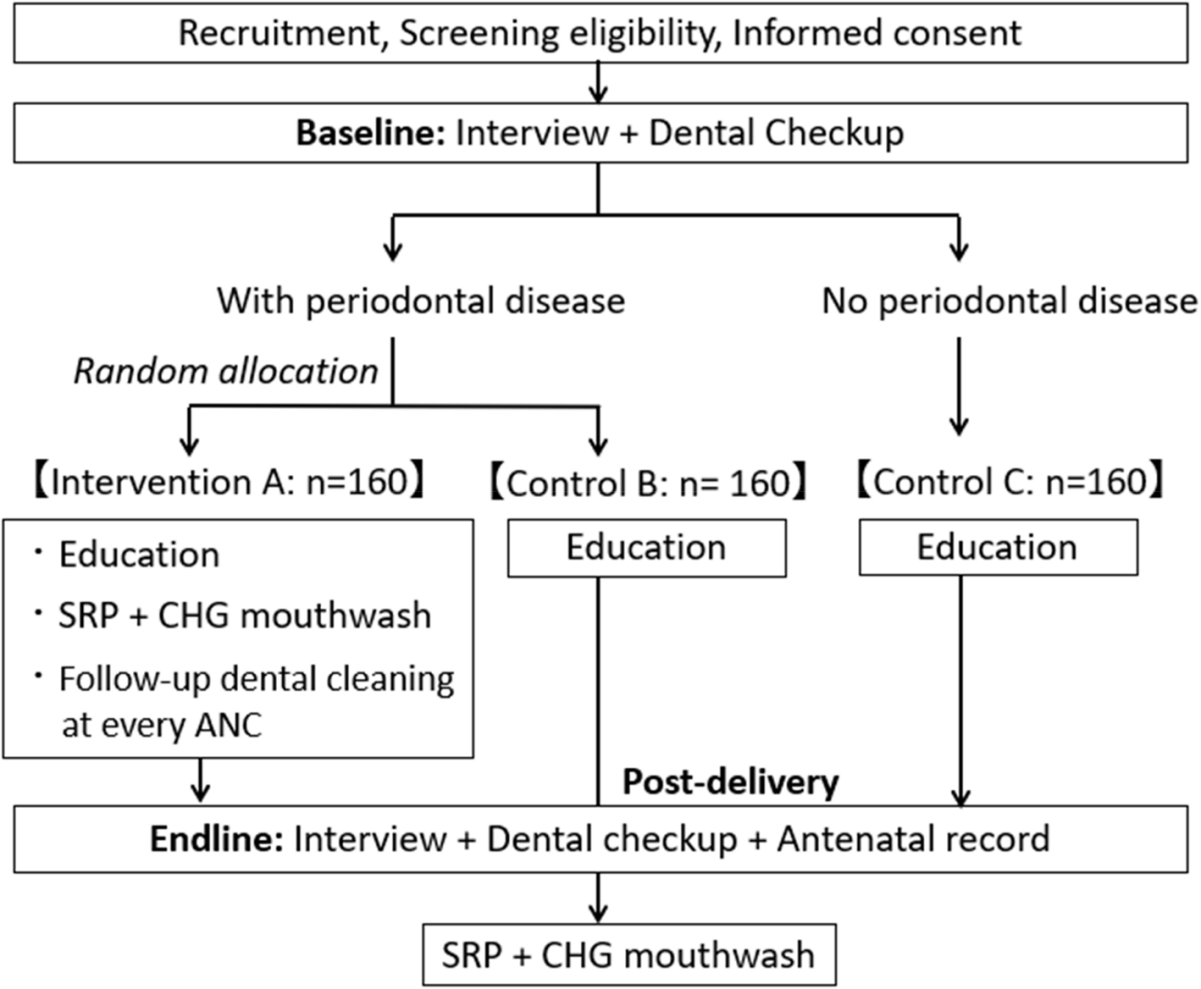
Flow chart of the study

**Fig. 2:**
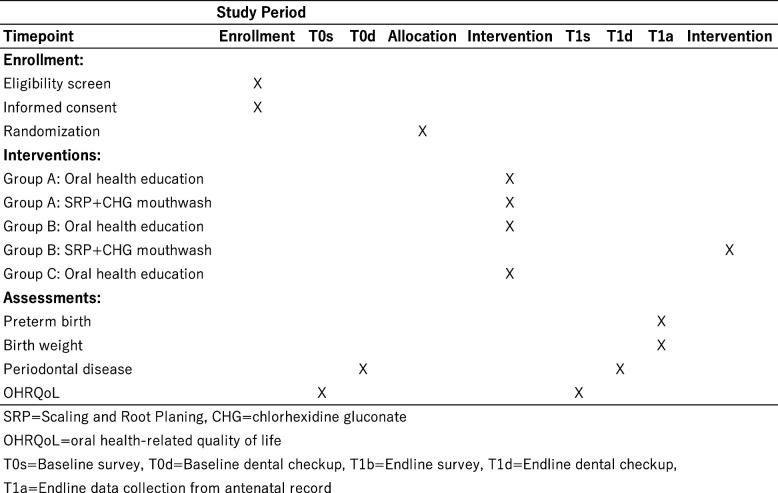
Schedule of enrolment, interventions, and assessments (SPIRIT flow diagram)

This study is a multi-center, three-arm RCT, consisting of one intervention group (group A) and two control groups (group B and C). The trial is designed in accordance with the Standard Protocol Items: Recommendations for Intervention Trials (SPIRIT) statement. Figure 1 shows a flow chart of the study design, including baseline and endline surveys, dental checkups, and intervention. Figure 2 summarizes the timeline of enrolment, interventions, and assessments, as per the SPIRIT flow diagram.

### Study sites

This trial will be conducted from April 2025 to September 2026 at two hospitals in Bangladesh: Holy Family Red Crescent Medical College Hospital (HFRC) in Dhaka and TMSS Medical College & Rafatullah Community Hospital (TMSS) in Bogura. Both the obstetrics and gynecology, and dental departments of each hospital will implement the interventions. Research coordinators from Grameen Communications will oversee overall coordination.

### Study population

Pregnant women attending antenatal checkups will be recruited from the obstetrics and gynecology departments at both hospitals, targeting approximately 225 participants per site. Enrollment will continue until 160 participants are allocated to each study group. Trained healthcare professionals, such as doctors, dentists, nurses, midwives, and dental assistants, who have completed a training program led by the research team, will assess eligibility, explain the study procedures, obtain informed consent, and conduct the intervention.

### Inclusion and exclusion criteria

Eligible participants will include pregnant women aged 18 years or older, with a gestational age of less than 20 weeks at recruitment. Women with multiple or high-risk pregnancies, as confirmed by attending healthcare professionals, will be excluded. High-risk conditions include diabetes, epilepsy, seizure disorders, and hypertension.

#### Hypothesis

We hypothesize that integrating an intervention targeting periodontal disease—comprising SRP and CHG mouthwash use—into the MNCH CoC will reduce PTB and LBW, decrease the prevalence of periodontal disease, and improve OHRQoL.

### Randomization and blinding

The trial includes three groups: group A (intervention) and groups B and C (control). All participants will first complete a face-to-face interview at the obstetrics and gynecology department following consent. They will then undergo a baseline dental checkup. Participants diagnosed with periodontal disease will be randomized to group A or B using blocked randomization via computer-generated sequence at each site. Those without periodontal disease will be assigned to group C.

Blinding will be maintained for the interviewers and data recorders, who will not be informed of group allocation. However, blinding will not be feasible for the dentists conducting dental checkups or for participants due to the nature of the intervention.

### Sample size calculation

Assuming a baseline PTB rate of 16.2% [25], and expecting the intervention to reduce this to 4.7% [32], a sample size of 128 per group is required to detect this difference with 80% power at a 5% significance level. Accounting for a 20% attrition rate, the total sample size is set at 480 (160 per group).

### Interventions

All participants will receive oral health education at baseline. The intervention being tested in this trial is the combination of SRP and the daily use of CHG mouthwash during pregnancy.

#### Oral health education

Immediately after the baseline survey and dental checkup, all participants will receive oral health education through a 5-min video and a culturally tailored handbook in Bengali. Two versions of the handbook, developed by the study’s dental experts, will be distributed: one for group A and another for groups B and C. Topics include proper tooth brushing, fluoride toothpaste use, and tongue cleaning [34–36]. Each handbook includes a daily self-monitoring chart designed to promote habit formation and support the maintenance of consistent oral hygiene behaviors throughout pregnancy. Group A’s version also includes CHG mouthwash usage instructions.

To increase accessibility and engagement, the video is accessible via QR code for viewing on personal or hospital-provided electronic devices.

#### Basic periodontal treatment and mouthwash use

Participants in group A will receive SRP and CHG mouthwash immediately after baseline dental checkups. Group B will receive the same intervention after the endline checkup. SRP procedures will take 10 to 15 min per patient. CHG use involves rinsing after morning and evening toothbrushing. Participants will log their daily hygiene practices in the provided handbook. Group A will receive follow-up dental cleaning using a toothbrush during subsequent antenatal visits.

### Data collection and study outcomes

The primary outcome is PTB (< 37 weeks gestation). Secondary outcomes include LBW (< 2500 g), periodontal disease, and OHRQoL.

#### Surveys

Face-to-face interviews at baseline will collect data on socio-demographic characteristics, obstetric history, oral care behaviors, OHRQoL, and lifestyle. An endline survey, conducted post-delivery before hospital discharge, will obtain data on oral health care behaviors, OHRQoL, and lifestyle.

A structured questionnaire was developed in English, translated into Bengali, and then back-translated into English. The accuracy of the translation and reliability of the contents were reviewed by two native Bengali speakers with expertise in MNCH and dentistry. A pre-test was conducted to identify any biases or ambiguities and to assess whether the questions are culturally appropriate.

##### Socio-demographic data

Socio-demographic data, including age, marital status, education level, employment status, family structure, and birth history, will be collected. Information on household assets, such as the ownership of durable goods, housing characteristics, and access to utilities, will also be collected to estimate household socioeconomic status by principal component analysis [37].

##### Oral health

Data on oral hygiene practices, especially tools, timing, and frequency of oral care, and knowledge of the epidemiology and prevention of periodontal disease will be collected. OHRQoL will be assessed via the Oral Health Impacts Profile (OHIP-14) [38, 39]. The profile consists of 14 questions in seven domains: functional limitation, physical pain, psychological discomfort, physical disability, psychological disability, social disability, and handicap. Responses are recorded, using a five-point Likert scale to record the responses: never = 1, hardly ever = 2, occasionally = 3, fairly often = 4, and very often = 5. The total score will be dichotomized into high versus low impact, using a median split.

The presence of periodontal disease symptoms will be assessed, using ten questions from a self-report check list developed by the Japanese Academy of Clinical Periodontology [40]. The questions include items related to bad breath, sticky mouth, bleeding during toothbrushing, swollen/drooping/bleeding gums, and problems with the teeth. The presence or absence of any symptoms will be included in further analysis.

##### Lifestyle

Data on lifestyle factors related to oral health, such as eating, smoking, and sleep habits, will be collected. At baseline, selected items from the Diet Quality Questionnaire (DQQ) [41] will also be administered to assess the intake of food items associated with an increased risk of periodontal disease. The DQQ consists of yes/no questions regarding the consumption of sentinel foods from 29 food groups consumed on the previous day. For this study, 16 food groups considered highly relevant to the development or prevention of periodontal disease have been selected, including (1) starchy staple foods, (2) vegetables, (3) fruits, (4) sweet foods, and (5) beverages.

To assess participants’ physical activity over the past 7 days, which has been shown to have a significant relationship with periodontal disease, the International Physical Activity Questionnaire short form (IPAQ-SF) [42] is included in the baseline survey. The IPAQ-SF comprises seven items covering four categories: vigorous-intensity activity, moderate-intensity activity, walking, and sitting. It also includes questions on the frequency and duration of physical activity. In the vigorous-intensity category, participants will be asked: “During the last 7 days, on how many days did you do vigorous physical activities like heavy lifting, digging, aerobics, running or fast bicycling?” and “How much time did you usually spend doing vigorous physical activities on one of those days?” Similar questions are asked about moderate-intensity physical activity (e.g., carrying light loads, jogging or bicycling), as well as for walking and sedentary behavior.

##### Delivery method

Information on the mode of delivery (e.g., vaginal delivery or caesarean section) and the associated decision-making process will be asked in the endline survey.

#### Dental checkups

Dental checkups (approximately 10 min per participant) will be conducted at both baseline and endline to collect dental data and diagnose periodontal disease [43]. A full-mouth examination will be performed to assess overall oral hygiene status, evaluating the condition of the teeth (sound, carious, treated, or missing), bleeding on probing (BOP), probing pocket depth (PPD), clinical attachment loss (CAL), plaque, calculus, tooth mobility, and oral dryness. BOP is defined as the presence or absence of bleeding during examination of the periodontal pocket. PPD refers to the distance from the gingival margin to the bottom of the pocket, while CAL is the distance from the cemento-enamel junction to the bottom of the pocket [44–46]. Oral dryness will be assessed using a classification scale based on the condition of the tongue mucosa, with higher grades indicating more severe dryness [47].

##### Diagnosis of periodontal disease

BOP, PPD, and CAL will be measured at six points on each tooth and used to diagnose periodontal diseases, including gingivitis and periodontitis. Gingivitis is diagnosed when BOP is present at 10% or more of the total number of periodontal sites examined. Periodontitis will be diagnosed if either of the following criteria is met: (1) interdental CAL is observed at ≥ 2 non-adjacent teeth, or (2) a buccal or palatal/lingual site exhibits a CAL of ≥ 3 mm and PPD of ≥ 4 mm at ≥ 2 teeth [45, 46].

Participants diagnosed with periodontal disease will be randomized into either the intervention group A or control group B. Participants without periodontal disease will be allocated to control group C.

#### Antenatal records

Data on birth outcomes, including gestational age at delivery and birth weight, will be extracted from antenatal records. Additional information will also be collected, including maternal height, pre-pregnancy body mass index, blood glucose level, pregnancy complications, and the number of antenatal checkups attended.

### Data management

At each hospital, survey data and antenatal records will be independently entered and electronically stored on password-protected servers, with access restricted to researchers listed in the approved IRB protocol. Dental data will initially be collected using paper-based forms and subsequently entered into electronic databases. All physical forms will be stored in a locked file cabinet in a locked office, accessible only to authorized researchers.

To ensure confidentiality and data security, each participant will be assigned a unique identification number, and all data will be anonymized accordingly. The master file linking participants’ names to their respective ID numbers will be stored in a password-protected folder on a secure computer at the Principal Investigator’s institution. All data analyses will be performed using de-identified data, with no access to personally identifiable information. In the event that a participant withdraws from the study, only the data collected up to the point of withdrawal will be included in the analyses.

Study findings will be disseminated through publications in peer-reviewed scientific journals and presentations at international conferences. The objective is to inform not only medical and academic communities, but also policymakers, in order to facilitate the potential integration of the intervention into institutional and national policies.

### Monitoring

The trial will be monitored on a daily basis by the research team, including the Principal Investigator, research coordinators, and other researchers listed on the IRB protocol. As the procedures involved—dental checkup and treatment—are considered to pose minimal risk to participants, including those with periodontal disease, a formal Data Monitoring Committee will not be established.

Nonetheless, the research team will address any issues that arise during the study to ensure adherence to ethical and regulatory standards. In the event of significant protocol modifications, such as changes to the inclusion/exclusion criteria or alterations to the primary outcome, the Principal Investigator will submit the proposed changes for review and approval by the Research Ethics Committee and the relevant hospital authorities at the study sites.

### Statistical analyses

The primary analysis will follow the intention-to-treat principle, whereby participants will be analyzed according to their assigned groups, regardless of adherence to CHG mouthwash use during pregnancy. Missing data will be handled using multiple imputation procedures available in the MI suite of Stata. To assess the robustness of the findings, sensitivity analyses will be conducted by comparing results from imputed and non-imputed datasets.

Data from the baseline and endline surveys, dental checkups, and antenatal records will be compared across groups to evaluate the effects of the intervention (SRP combined with CHG mouthwash use) on the primary (PTB) and secondary outcomes (LBW, periodontal disease, and OHRQoL). Initial analyses will include descriptive statistics for participants’ socio-demographic and other basic characteristics, followed by bivariate analyses to examine differences in baseline variables between groups A and B, and between groups B and C.

To estimate the causal effect of the intervention on birth outcomes, comparisons will be made between groups A and B. To investigate whether previously reported associations between periodontal disease and adverse birth outcomes hold in our population, groups B and C will be compared.

For multivariable analyses, two types of regression models will be employed. (1) Multiple regression models will be used to evaluate outcomes measured only at endline. (2) Mixed-effect regression models will be applied for outcomes assessed at both baseline and endline, accounting for potential intra-individual serial correlations in the residual.

Specifically, to assess the effectiveness of the intervention on birth outcomes (PTB and LBW), a multiple regression model will be used, with intervention status (group A or B) as the main predictor, adjusting for potential covariates (socio-demographic characteristics, lifestyle, pregnancy-related characteristics, and pregnancy outcomes). For oral health outcomes (periodontal disease and OHRQoL), mixed-effects regression models will be used, including intervention status (group A or B), time point (baseline or endline), and their interaction terms, while adjusting for potential covariates.

To evaluate the association between baseline periodontal disease and birth outcomes in groups B and C, a multiple regression model will be employed, with baseline periodontal status (group B or C) as the exposure, adjusting for potential confounders (socio-demographic characteristics and lifestyle at baseline). To assess change in periodontal status between baseline and endline in groups B and C, another multiple regression model will be used with similar covariate adjustments.

To evaluate the association of baseline periodontal diseases and OHRQoL in groups B and C, mixed-effect regression models will be fitted, including baseline periodontal status (group B or C), time point (baseline or endline), and their interaction term, adjusting for the possible confounding factors listed above.

Statistical significance will be set at a *p*-value of < 0.05. All statistical analyses will be conducted using IBM SPSS Statistics version 26.0 (IBM Corp, Armonk, NY, USA) or Stata version 16.0 (StataCorp LLC, College Station, TX, USA).

## Discussion

This protocol describes a RCT to be conducted at two hospitals in Bangladesh, aiming to evaluate the effectiveness of integrating an oral health intervention into the MNCH CoC for the prevention of adverse birth outcomes. Specifically, this study investigates whether a combination of basic periodontal treatment (SRP) and the continued use of CHG mouthwash during pregnancy can prevent PTB and LBW. Although the association between oral hygiene and various systemic conditions has garnered increasing attention, the effective oral care methods for preventing adverse birth outcomes remain unclear. Most previous interventional studies have demonstrated that SRP alone is insufficient to significantly reduce the risk of PTB and LBW [29, 30].

At present, strategies to manage periodontal disease during pregnancy and promote oral health care for mothers and children have not been incorporated into the MNCH CoC framework or the World Health Organization’s guidelines for antenatal and postnatal care. In light of the increasing recognition of the importance of maternal oral hygiene, there is a need for a more proactive and sustained approach to oral health. The effectiveness of such an approach may be particularly evident in resource-limited settings, where MNCH indicators remain suboptimal.

This study has some limitations. First, the sample will consist solely of pregnant women in Bangladesh, recruited from two regions (Dhaka and Bogura), which may limit the generalizability of the findings due to potential sampling bias. Second, because of the nature of the intervention, blinding of the dentists performing the baseline dental assessments and the participants will not be feasible. To minimize the risk of assessment bias, endline dental checkups will be performed by independent dentists who were not involved in the baseline dental checkups.

Despite these limitations, demonstrating the effectiveness of this oral health intervention in a real-world setting could provide valuable insights into the potential contribution of oral care to improving MNCH outcomes, particularly in low-resource environments.

### Trial status

Protocol version 1 (December 2024). Recruitment began on April 27, 2025, and we anticipate concluding the recruitment phase by December 2025. Overall data collection is expected to end in September 2026.

## Supplementary Information

Supplementary Material 1. SPIRIT checklist.

## Data Availability

The datasets used and/or analyzed during the study will be available from the corresponding author on reasonable request.

## Abbreviations

CHG: Chlorhexidine gluconate
CoC: Continuum of care
DQQ: Diet Quality Questionnaire
HFRC: Holy Family Red Crescent Medical College Hospital
LBW: Low birth weight
MNCH: Maternal, Neonatal, and Child Health
OHRQoL: Oral health-related quality of life
PTB: Preterm birth
RCT: Randomized controlled trial
SRP: Scaling and root planing
TMSS: TMSS Medical College & Rafatullah Community Hospital
